# Oxidative stress in the development, maintenance and resolution of paclitaxel-induced painful neuropathy

**DOI:** 10.1016/j.neuroscience.2016.06.050

**Published:** 2016-10-01

**Authors:** Natalie A. Duggett, Lisa A. Griffiths, Olivia E. McKenna, Vittorio de Santis, Nutcha Yongsanguanchai, Esther B. Mokori, Sarah J.L. Flatters

**Affiliations:** Wolfson Centre for Age-Related Diseases, Institute of Psychiatry, Psychology and Neuroscience, King’s College London, London SE1 1UL, UK

**Keywords:** BSA, bovine serum albumin, CGRP, calcitonin gene-related peptide, DAPI, 4',6-diamidino-2-phenylindole, DMSO, dimethyl sulfoxide, DRG, dorsal root ganglia, EDTA, ethylenediaminetetraacetic acid, FBS, foetal bovine serum, FITC, fluorescein isothiocyanate, GFAP, glial fibrillary acidic protein, GPx, glutathione peroxidase, IB4, isolectin-B4, MEM, minimum essential media, NGS, normal goat serum, PBS, phosphate-buffered saline, PFA, paraformaldehyde, ROS, reactive oxygen species, SOD, superoxide dismutase, mitochondria, chemotherapy, Taxol, chemotherapy-induced neuropathy, neurotoxicity, reactive oxygen species

## Abstract

Paclitaxel is a first-line chemotherapeutic with the major dose-limiting side effect of painful neuropathy. Previous preclinical studies indicate mitochondrial dysfunction and oxidative stress are associated with this disorder; however no direct assessment of reactive oxygen species (ROS) levels and antioxidant enzyme activity in sensory neurons following paclitaxel has been undertaken. As expected, repeated low doses of systemic paclitaxel in rats induced long-lasting pain behaviour with a delayed onset, akin to the clinical scenario. To elucidate the role of ROS in the development and maintenance of paclitaxel-induced painful neuropathy, we have assessed ROS and antioxidant enzyme activity levels in the nociceptive system *in vivo* at three key behavioural time-points; prior to pain onset (day 7), peak pain severity and pain resolution. In isolated dorsal root ganglia (DRG) neurons, ROS levels were unchanged following paclitaxel-exposure *in vitro* or *in vivo*. ROS levels were further assessed in DRG and spinal cord *in vivo* following intrathecal MitoTracker®RedCM-H_2_XRos administration in paclitaxel-/vehicle-treated rats. ROS levels were increased at day 7, specifically in non-peptidergic DRG neurons. In the spinal cord, neuronally-derived ROS was increased at day 7, yet ROS levels in microglia and astrocytes were unaltered. In DRG, CuZnSOD and glutathione peroxidase (GPx) activity were increased at day 7 and peak pain time-points, respectively. In peripheral sensory nerves, CuZnSOD activity was increased at day 7, and at peak pain, MnSOD, CuZnSOD and GPx activity were increased. Catalase activity was unaltered in DRG and saphenous nerves. These data suggest that neuronally-derived mitochondrial ROS, accompanied with an inadequate endogenous antioxidant enzyme response, are contributory factors in paclitaxel-induced painful neuropathy.

## Highlights

• ROS levels assessed in peripheral and central sensory neurons following paclitaxel.
• Increased ROS levels seen in non-peptidergic neurons prior to paclitaxel-induced pain.
• Elevated ROS levels in spinal neurons, but not microglia/astrocytes, after paclitaxel.
• Assayed activity of main antioxidant enzymes during paclitaxel-evoked pain timecourse.
• Inadequate antioxidant response suggests elevated ROS sustains paclitaxel-evoked pain.

## Introduction

Paclitaxel (Taxol®) is a taxane-derived chemotherapeutic and first-line treatment for solid tumours. Its primary anti-cancer action occurs via disruption of the mitotic spindle and microtubule dynamics causing apoptosis (Fuchs and Johnson, 1978, Rowinsky et al., 1988, Jordan et al., 1993). The major dose-limiting side effect of paclitaxel treatment is painful peripheral neuropathy, which is predominantly sensory and increases with cumulative dosing (Postma et al., 1995). Affected patients typically report bilateral numbness, tingling, spontaneous pain and evoked pain to mechanical and cold stimuli in the hands and/or feet (Forsyth et al., 1997, Dougherty et al., 2004, Boyette-Davis et al., 2013). Paclitaxel-induced neuropathy can persist for months or years following cessation of paclitaxel (van den Bent et al., 1997, Dougherty et al., 2004, Boyette-Davis et al., 2013). At present, there are no treatments to prevent the development of paclitaxel-induced neuropathy and/or reverse it when established. Therefore, the emergence of painful neuropathy during paclitaxel therapy often results in the discontinuation of otherwise successful chemotherapy, thus impacting on both the quality of life (Tofthagen, 2010) and survival of cancer patients.

A rat model of paclitaxel-induced painful neuropathy, using low doses of clinically-formulated paclitaxel, mimics the scenario of multiple non-consecutive systemic injections (akin to treatment cycles), evoking mechanical/cold hypersensitivities, with similar time-courses to those seen in patients (Flatters and Bennett, 2004, Flatters and Bennett, 2006, Fidanboylu et al., 2011, Griffiths and Flatters, 2015). Using this model, an extensive electron microscopy study identified paclitaxel-induced changes in the mitochondria of sensory axons in saphenous nerves which correlated with development and maintenance of paclitaxel-induced pain behaviour (Flatters and Bennett, 2006).

Several reports have explored the contributory role of reactive oxygen species (ROS) *in vivo* to paclitaxel-induced pain behaviours using pharmacological ROS scavenging agents. Phenyl-N-tert-butylnitrone (PBN), a nonspecific ROS scavenger, inhibited development and reversed established paclitaxel-induced pain behaviours (Kim et al., 2010, Fidanboylu et al., 2011). Peroxynitrite decomposition catalysts have also been shown to prevent the development and reversed established paclitaxel-induced mechanical hypersensitivity (Doyle et al., 2012). Systemic acetyl-l-carnitine (ALC) administration prevented the development of paclitaxel-induced mechanical hypersensitivity (Flatters et al., 2006) and the paclitaxel-evoked increase of atypical mitochondria in C-fibres of the saphenous nerve (Jin et al., 2008). However, there is no direct evidence demonstrating *in vivo* paclitaxel treatment increases ROS production *in vivo*. *In vitro* investigations have shown increased ROS following paclitaxel exposure in isolated rat liver mitochondria (Varbiro et al., 2001), human breast (Fawcett et al., 2005, Alexandre et al., 2007) and bladder (Ramanathan et al., 2005) cancer cell lines, but whether paclitaxel can increase ROS in sensory neurons specifically, *in vitro* and/or *in vivo*, remains to be clarified.

Several endogenous antioxidant enzymes work to control ROS to prevent damage. The superoxide dismutase (SOD) family consists of three enzymes; mitochondrial MnSOD, predominately cytoplasmic CuZnSOD, and extracellular, non-neuronal derived ECSOD (Okado-Matsumoto and Fridovich, 2001, Zelko et al., 2002). MnSOD and CuZnSOD rapidly scavenge and dismutate superoxide, acting as a first line of defence against electron transport chain-derived oxidative stress (Zelko et al., 2002, Candas and Li, 2014). Other key facets of the ROS management response are glutathione peroxidase (GPx) and peroxisome-based catalase. Their discrete subcellular locations allow rapid, specific hydrogen peroxide management (Weydert and Cullen, 2010). The activity of endogenous antioxidant enzymes – SOD, GPx and catalase – likely determine the consequence of ROS and an upregulation in their activity could indicate increased ROS.

Given our previous findings (Flatters and Bennett, 2006, Fidanboylu et al., 2011), here we aim to elucidate the putative contributory role of ROS in development and maintenance of paclitaxel-induced painful neuropathy. We sought to determine whether ROS levels changed in sensory neurons in response to paclitaxel exposure *in vitro* or *in vivo*. We have examined ROS levels *in vivo* at key junctions of nociceptive signalling integration – the dorsal root ganglia (DRG) and spinal cord – prior to, during, and at the resolution of the paclitaxel-induced pain behaviour. Furthermore, we have assessed the activity of the major antioxidant enzymes – MnSOD, CuZnSOD, GPx & catalase – in peripheral sensory nerves and DRG at these three critical time points. Therefore, through an extensive series of experiments, we have addressed where ROS levels are altered in the nociceptive system and the status of the antioxidant response at these sites, in correlation to the time-course of paclitaxel-induced painful neuropathy. Data from these studies were previously presented in abstract form (Griffiths et al., 2012, Duggett et al., 2015).

## Experimental procedures

### Behavioural assessment and drug administration

Adult male Sprague–Dawley rats (180–220 g; Harlan) were housed in cages of 3–4 with sawdust bedding and environmental enrichment materials*,* in a climate-controlled environment with a 12 h light/dark cycle (lights on at 7 am)*.* Food and water were freely available. All procedures were conducted in strict accordance with the UK Animals (Scientific Procedures) Act, 1986 and the IASP ethical guidelines (Zimmermann, 1983). The protocol was approved by the Ethics Review Panel of King’s College London and conducted under the UK Home Office project license 70/8015. As previously described (Fidanboylu et al., 2011, Griffiths and Flatters, 2015), animals were habituated to the testing environment and mechanical hypersensitivity was assessed by withdrawal responses to von Frey filaments with bending forces of 4g, 8g and 15g. Three baseline measurements were taken prior to paclitaxel/vehicle administration and mechanical hypersensitivity was measured at 1–3 week intervals until the paclitaxel-induced pain syndrome resolved. Three critical time-points of paclitaxel-induced mechanical hypersensitivity were investigated in these studies: day 7 – 24 h after the last injection of paclitaxel, prior to emergence of mechanical hypersensitivity; day 23–31 – peak of mechanical hypersensitivity (von Frey responses recorded as ⩾2.5-fold higher than baseline responses); day 173–220 – resolution of mechanical hypersensitivity (return to individual baseline responses observed on two separate occasions). Sample sizes were based on the number of animals used in a given experiment.

The clinical formulation of 6 mg/ml Paclitaxel Solution for Infusion (Actavis Ltd, Barnstaple, Devon, UK) was diluted with 0.9% sterile saline (Fresenius Kabi, Runcorn, Cheshire, UK) to achieve a 2-mg/ml solution. A vehicle solution of 1:1 cremophor EL and ethanol, plus 2 mg/ml sodium citrate, replicated the clinical formulation of paclitaxel. For vehicle administration; one part vehicle solution was diluted with two parts 0.9% sterile saline. Animals received intraperitoneal 2 mg/kg paclitaxel or equivalent volume of vehicle solution on four alternate days (0, 2, 4, 6), as previously described (Flatters and Bennett, 2006).

### Isolation of dorsal root ganglia (DRG) neurons

DRG were dissected bilaterally starting from L6/L5, proceeding rostrally with 10–14 DRG harvested from each animal. Further dissection removed the ventral root, and partially removed the dura mater/dorsal root. DRG were then transferred to fresh MEM (Sigma, Gillingham, Dorset, UK) containing 1% penicillin/streptomycin (Invitrogen, Paisley, Renfrewshire, UK), 2.5 mg/ml collagenase (type IV, Worthington Biochemicals, Lakewood, New Jersey, USA), and incubated at 37 °C for 3 h. DRG were triturated and trypsin (0.25 mg/ml) added for 10–20 min at 37 °C. A single-cell suspension was diluted with MEM + 10% FBS, 1% P/S, and centrifuged at 1000 rpm for 5 min. The pellet was resuspended in MEM +1% P/S, 10% FBS, 0.1 mg/ml DNase I (Worthington Biochemicals, Lakewood, New Jersey, USA), pipetted onto a 15% BSA (Sigma, Gillingham, Dorset, UK) cushion in MEM, and centrifuged for 10 min at 1000 rpm. The debris/myelin layer formed at the solution interface was discarded before removing media to leave a cell pellet. This was resuspended in MEM +1% P/S, 10% FBS, 0.1% cytosine arabinoside before plating.

### *In vitro* application of fluorescent mitochondrial probes

In all experiments, dissociated DRG cells were plated onto poly-d-lysine-coated glass coverslips in humidified 4-well plates and kept at 37 °C, 5% CO_2_ overnight. Coverslips were exposed to 250 ng/ml paclitaxel (prepared from clinically-formulated 6 mg/ml Paclitaxel with sterile saline/MEM) or equivalent vehicle solution for 3 h at 37 °C, 5% CO_2_. In separate experiments, DRG neurons were isolated from paclitaxel-/vehicle-treated rats at day 7 (*n* = 8 animals/group), peak pain (days 23–31, *n* = 7 animals/group) and resolution of pain (days 173–194, *n* = 7 animals/group).

Two mitochondrial-targeted reduced probes were used to identify ROS; MitoTracker® Red CM-H_2_XRos (MTRed; Molecular Probes, Thermo Fisher Scientific, M7513, Loughborough, Leicestershire, UK) and MitoSOX™ Red (MitoSOX; Molecular Probes, Thermo Fisher Scientific, M7514, Loughborough, Leicestershire, UK). MTRed fluoresces following oxidation by all ROS species, whereas MitoSOX fluorescence is predominantly via oxidation by superoxide radicals. MTRed/MitoSOX were reconstituted in 1:1 DMSO & 0.9% sterile saline, followed by further dilution in MEM. Coverslips were exposed to 5 μM MTRed or 1 μM MitoSOX for 15 min, at 37 °C, 5% CO_2_, in the dark. Solutions were removed and coverslips were fixed in cold 4% PFA for 5 min, rinsed with phosphate-buffered saline (PBS), then dH_2_O, and mounted onto slides using Vectashield Antifade Mounting Medium (Vectashield; Vector Laboratories, Peterborough, Cambridgeshire, UK). To show co-localisation of MTRed and MitoSOX probes in mitochondria in these DRG cell preparations, coverslips were simultaneously exposed to 200 nM MTGreen and 5 μM MTRed or 1 μM MitoSOX as described above. Coverslips were then fixed, washed and mounted onto slides using Vectashield Antifade Mounting Medium with DAPI (Vectashield + DAPI; Vector Laboratories, Peterborough, Cambridgeshire, UK).

Coverslips were imaged using a fluorescence microscope (AxioPlan2; Zeiss, Jena, Germany) with a Plan NeoFluar 20×/0.50 objective lens. For cells co-labelled with MTGreen, images were taken with a fluorescence microscope with ApoTome attachment (AxioPlan2; Zeiss, Jena, Germany) and a Plan Apochromat 40×/0.95 objective lens. Using Adobe Photoshop CS5 Extended, diameter and fluorescence intensity of each DRG neuron was measured. DRG neurons were easily distinguished from non-neuronal cells due to their greater fluorescence and large, rounded appearance. DRG neurons were categorised based on diameter: small (10–25 μm), medium (25–50 μm) or large (>50 μm). The number of neurons measured in each category is indicated in each figure. Sample sizes in all analyses are based on the number of animals used in each experiment. Mean fluorescence intensity of small, medium and large neurons was determined for each animal, then mean ± SEM calculated for the neuronal size category, from each group of animals used.

MTRed fluorescence intensity of DRG neurons from paclitaxel-treated animals was expressed as a percentage of the MTRed fluorescence intensity of DRG neurons from vehicle-treated animals within each harvest (consisting of one vehicle-treated and one paclitaxel-treated rat; *n* = 7–8 animals per group). This within-experiment normalisation controlled for variability between different lot numbers of MTRed/MitoSOX and microscope bulbs used during the experiments. Therefore, paired data analysis was used for these experiments. All image analysis was performed under blind conditions.

### *In vivo* application of fluorescent mitochondrial probes

Pilot studies determined that *in vivo* application of these probes to peripheral sensory nerves via epineural injection did not allow sufficient spread and permeation for adequate analysis of ROS in peripheral nerves. Under isoflurane anaesthesia, paclitaxel-/vehicle-treated rats received intrathecal (IT) injections of 90 μl of 100 μM MTRed (reconstituted with 1:1 DMSO and 0.9% sterile saline) or equivalent vehicle. Entrance to the intrathecal space was identified by tail movement. Pilot studies using MitoTracker® Red CMXRos (Molecular Probes, Thermo Fisher Scientific, Loughborough, Leicestershire, UK) a non-reduced purple-coloured probe, determined optimal injection site (between L5/L6 vertebrae) and volume required to ensure sufficient spread within the CSF to L4/L5 spinal cord. Five to six hours following IT injections, rats were overdosed with pentobarbital and transcardially perfused with 0.9% saline containing heparin, followed by cold 4% PFA. Vertebral columns were removed and post-fixed in 4% PFA overnight at 4 °C. L4/L5 DRG/spinal cord were dissected and immersed in 30% sucrose solution, at 4 °C for 3–5 days. Tissues were then embedded in OCT and stored at −80 °C until cryosectioning.

### *In vivo* ROS levels in DRG neurons

Sixteen micrometers sections of L4/L5 DRG were cut onto slides and coverslipped using Vectashield + DAPI. Mosaix montages of DRG sections were created using a fluorescent microscope (AxioPlan 2; Zeiss, Jena, Germany) with a Plan Apochromat 20×/0.75 objective lens and Texas Red/UV filters, for MTRed/DAPI-stained nuclei, respectively. The same exposure time for Texas Red filter was used for all images. Neuronal cell bodies with a round, central nucleus were selected using AxioVision LE software (v.4.8) to measure fluorescence intensity and diameter. Neuronal cell bodies were categorised as small (10–23 μm diameter), medium (23–32 μm) or large (>32 μm). The mean MTRed fluorescence of small, medium and large neurons was determined for individual animals (from three non-consecutive DRG sections per animal). The mean ± SEM MTRed fluorescence was calculated for each neuronal category for paclitaxel-/vehicle-treated rats at each time point (*n* = 6 animals/group). Any auto-fluorescence of DRG neurons (using DRG sections following vehicle IT injections) could not be visualised with the chosen exposure time and was concluded to be minimal. Furthermore, analysis of spinal cord sections (see below) from rats that received vehicle IT injections, showed no difference in autofluorescence between paclitaxel-/vehicle-treated rats. All analyses were performed under blind conditions.

Sixteen micrometers L4/5 DRG sections were blocked for one hour at RT in PBS containing 3% normal goat serum (NGS), 0.3%Triton-X-100, 0.01% sodium azide. Calcitonin gene-related peptide (CGRP) or isolectin-B4 (IB4) were used to detect peptidergic or non-peptidergic neurons, respectively. DRG sections were incubated at 4 °C overnight in the dark, with mouse anti-CGRP (1:2000; Sigma, C7113, Gillingham, Dorset, UK) or IB4 (1:100; Sigma, L3759, Gillingham, Dorset, UK), in PBS containing 0.1% Triton-X-100 (PBS-T). Following three 10-min PBS washes, sections were exposed to goat anti-mouse FITC (for CGRP, 1:400; Jackson ImmunoResearch Laboratories, West Grove, Pennsylvania, USA) or ExAvidin conjugated with FITC (for IB4, 1:400; Stratech, Newmarket, Suffolk, UK) in PBS-T, for 90 min at RT, in the dark. Sections had three 10-min PBS washes and then coverslipped with Vectashield + DAPI. A three-channel Mosaix montage of the entire DRG section was taken using a Plan Apochromat 20×/0.75 objective lens and Texas Red/FITC/UV filters for MTRed/CGRP or IB4/DAPI-stained nuclei, respectively. The same exposure time for Texas Red filter was used for all images. CGRP-/IB4-positive neurons were selected to measure MTRed fluorescence and diameter, with mean MTRed fluorescence determined for each animal (from measurements on one CGRP-labelled section and 1–2 non-consecutive IB4-labelled sections per animal). The mean ± SEM MTRed fluorescence was calculated for each neuronal category, for paclitaxel-/vehicle-treated rats at each time point (*n* = 5–6 animals/group). All analyses were performed under blind conditions.

### *In vivo* ROS levels in the spinal cord

Ten micrometers sections of L4/L5 spinal cord were cut, mounted and coverslipped using Vectashield. Images of the dorsal horn were taken using a fluorescent microscope with Plan NeoFluar 20×/0.50 objective lens and Texas Red filter. One image was taken per section and the same exposure time used for all images. Using AxioVision, an area (132,521.38 μm^2^) of laminae I-III was selected to measure the mean fluorescence intensity. Measurements on sections from rats that received IT vehicle (DMSO & saline) injections showed similar average background-/auto-fluorescence between paclitaxel- & vehicle-treated rats. Thus, an average background fluorescence value was calculated at each time point and subtracted from the MTRed fluorescence measured in each MTRed section at that time point. The average MTRed fluorescence was calculated for each animal from four to nine non-consecutive sections (two rats at pain resolution two sections/animal analysed). *n* = 6–10 for paclitaxel-/vehicle-treated rats at each time-point. All analyses were performed under blind conditions.

ROS levels in spinal neurons, microglia and astrocytes were examined in paclitaxel-/vehicle-treated rats at day 7. 10 μm spinal cord sections were blocked for 1 h at RT in PBS containing 3% NGS, 0.3% Triton-X-100, 0.01% sodium azide. DRG sections were incubated with NeuN (neuronal marker, 1:100 clone-A60 Millipore, MAB377, Watford, Hertfordshire, UK); ionized calcium binding adaptor molecule-1 (IBA-1, microglia marker, 1:1000 WAKO, 019-19741); or glial fibrillary acidic protein (GFAP, astrocyte marker, 1:5000 Millipore, MAB360, Watford, Hertfordshire, UK), in PBS-T, at 4 °C overnight in the dark. Following three 10-min PBS washes, sections were exposed to 1:400 goat anti-mouse or 1:400 goat anti-rabbit FITC secondary antibodies (Jackson ImmunoResearch Laboratories, West Grove, Pennsylvania, USA) in PBS-T for 90 min at RT in the dark. Finally, sections had three 10-min PBS washes and coverslipped with Vectashield. A two-channel Mosaix montage of laminae I–III of each section was taken using a Plan Apochromat 20×/0.75 objective lens and Texas Red/FITC filters for MTRed/NeuN;IBA-1;GFAP, respectively. Exposure times were kept consistent across slides.

For neuronal analysis; NeuN-positive cell bodies were selected in laminae I–III using AxioVision and MTRed fluorescence measured. The mean neuronal MTRed fluorescence was calculated for each animal from three to seven sections; *n* = 5–7 rats/group. A total of 4795 and 5474 neurons were analysed from vehicle- and paclitaxel-treated rats, respectively. For microglial analysis; using AxioVision, an area (132,521.38 μm^2^) of laminae I–III was selected and MTRed fluorescence in IBA-1-positive cell bodies within this area measured. The mean microglial MTRed fluorescence was calculated for each animal from four to six sections; *n* = 4 rats/group. For astrocytic analysis: using Axiovision, an area (132,521.38 μm^2^) of laminae I–III was selected. Due to the density/morphology of astrocytes, individual astrocytic cell bodies could not be identified. ImageJ (NIH) plugin, Colocalization Finder, was used to highlight pixels labelled with both MTRed and GFAP, giving a percentage of co-staining. All analyses were performed under blind conditions.

### In-gel activity assays of endogenous antioxidant enzymes

L4/5 DRG and saphenous nerves were harvested from vehicle- and paclitaxel-treated animals at the three time-points and flash frozen. Tissue was prepared as described (Weydert and Cullen, 2010) and methodology for in-gel activity assays was based on the methods described here (Woodbury et al., 1971, Sun et al., 1988, Weydert and Cullen, 2010). In brief, 12% or 8% 1.5 mm separating acrylamide gels were prepared, and run for 1 h at 4 °C using pre-electrophoresis buffer (190 mM Tris-Base, 1 mM disodium EDTA, pH 8.8). Samples were prepared 1:1 with loading buffer (250 mM Tris, 4 mM EDTA, 50% glycerol, 0.2% bromophenol blue).DRG (100mg) protein was used for SOD gels. In all other instances, 50 μg protein was used. Following sample loading, gels were run for three hours at 4 °C in pre-electrophoresis buffer. Gels were run for approx. 4 h at 4 °C in electrophoresis buffer (50 mM Tris-Base, 0.3 M glycine, 2 mM disodium EDTA, pH 8.3). Paclitaxel and vehicle samples from the same time point were run on the same gel. After staining, gels were thoroughly washed in dH_2_O and scanned using an Epson V370 scanner. Gels stained for MnSOD or total SOD (two gels run for each time point) as previously described (Weydert and Cullen, 2010). Gels stained for GPx or catalase as outlined (Woodbury et al., 1971, Sun et al., 1988, Weydert and Cullen, 2010). Staining techniques produced variable colour intensities; however this was controlled by running all vehicle and paclitaxel samples within the same gel. All gels were scanned as black and white images, bands were then selected and analysed in ImageJ. Band densities were normalised to adjacent background areas, with activity normalised to total protein loaded per well. *n* = 3–6 paclitaxel-/vehicle-treated rats per time-point.

All statistical analysis was conducted on raw data (not percentages) using GraphPad Prism 6 or GraphPad InStat 3 for Windows. Specific tests used are as indicated in figure legends. Statistical significance was accepted at *p* < 0.05. No further distinction was made when *p* < 0.01 or *p* < 0.001 and is denoted on figures as *p* < 0.05.

## Results

**Fig. 1:**
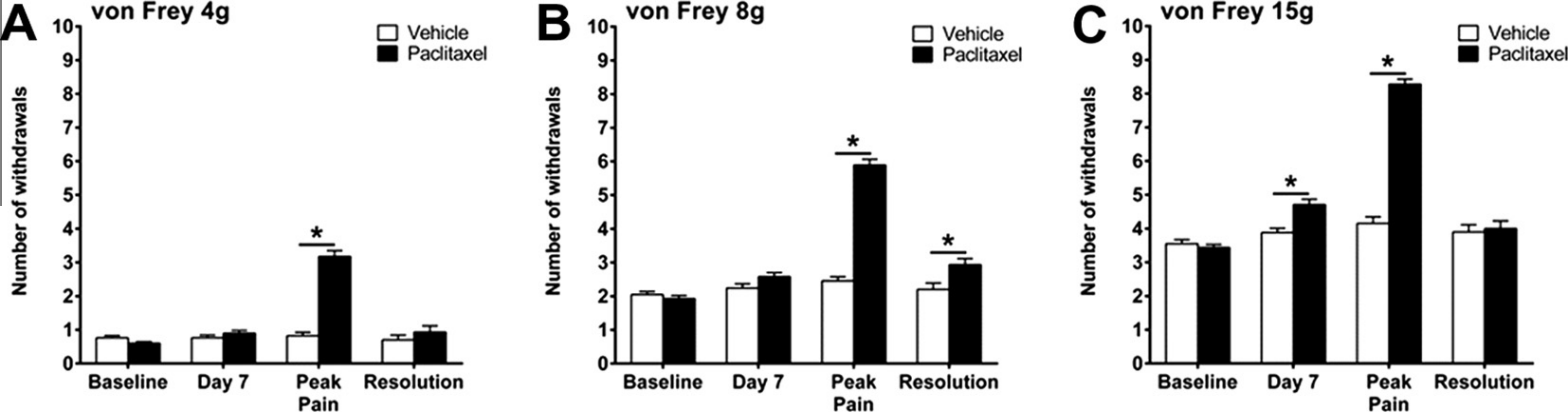
Time course of paclitaxel-induced mechanical hypersensitivity. Graphs show the mean ± SEM of the number of withdrawal responses to (A) 4g, (B) 8g and (C) 15g von Frey filaments at; baseline, day 7, peak pain (day 23–31) and pain resolution (day 173–220), following paclitaxel/vehicle administration at days 0, 2, 4 and 6. ^*^p < 0.05, two-tailed multiple comparison unpaired *t*-tests with Holm-Sidak correction. Day 7 *n* = 108 vehicle, *n* = 114 paclitaxel; peak pain *n* = 73 vehicle, *n* = 75 paclitaxel; resolution *n* = 30 vehicle, *n* = 30 paclitaxel. NB: These data are compiled from several cohorts of animals used to generate tissues for these studies. Data from each individual cohort show no significant difference in mechanical hypersensitivity, between vehicle and paclitaxel-treated animals, at day 7 and pain resolution.

We have consistently found that four systemic low-dose (2 mg/kg) injections of paclitaxel administered on days 0, 2, 4 & 6, evokes mechanical hypersensitivity with a gradual onset. Paclitaxel-induced mechanical hypersensitivity takes several weeks to reach its peak (approx. day 26), remains elevated for a few months and resolves approximately 6 months following the initial paclitaxel injection. In these studies, we examined tissues at three key time-points within this time-course; (1) day 7 – approx. 24 h after the last paclitaxel administration, prior to the onset of pain behaviour; (2) day 23–31 – peak of paclitaxel-induced pain behaviour; (3) day 173–220 – resolution of paclitaxel-induced pain behaviour. The day of harvest for each rat was dictated by the behavioural phenotype compared to individual baseline responses. Pain resolution was variable within cohorts. Fig. 1 illustrates the pain phenotype present prior to tissue harvesting for these *ex vivo* studies. As expected, we observed a ⩾2.5-fold significant increase in paw withdrawal responses to von Frey 4g, 8g and 15g stimulation at the peak of paclitaxel-induced mechanical hypersensitivity. In individual cohorts of animals, significant differences in mechanical hypersensitivity were not seen between paclitaxel- and vehicle-treated rats at day 7 and resolution of pain time-points. However, when these data were collated, small (less than one withdrawal) but statistically significant increases in responses to von Frey 8g at pain resolution (Fig.1B) and to von Frey 15g at day 7 (Fig.1C) were seen in paclitaxel-treated rats. It is highly likely that statistical significance observed at these time points is due to the high n numbers following data collation, day 7 – *n* = 108–114, pain resolution – *n* = 30, rather than a biological effect.

**Fig. 2:**
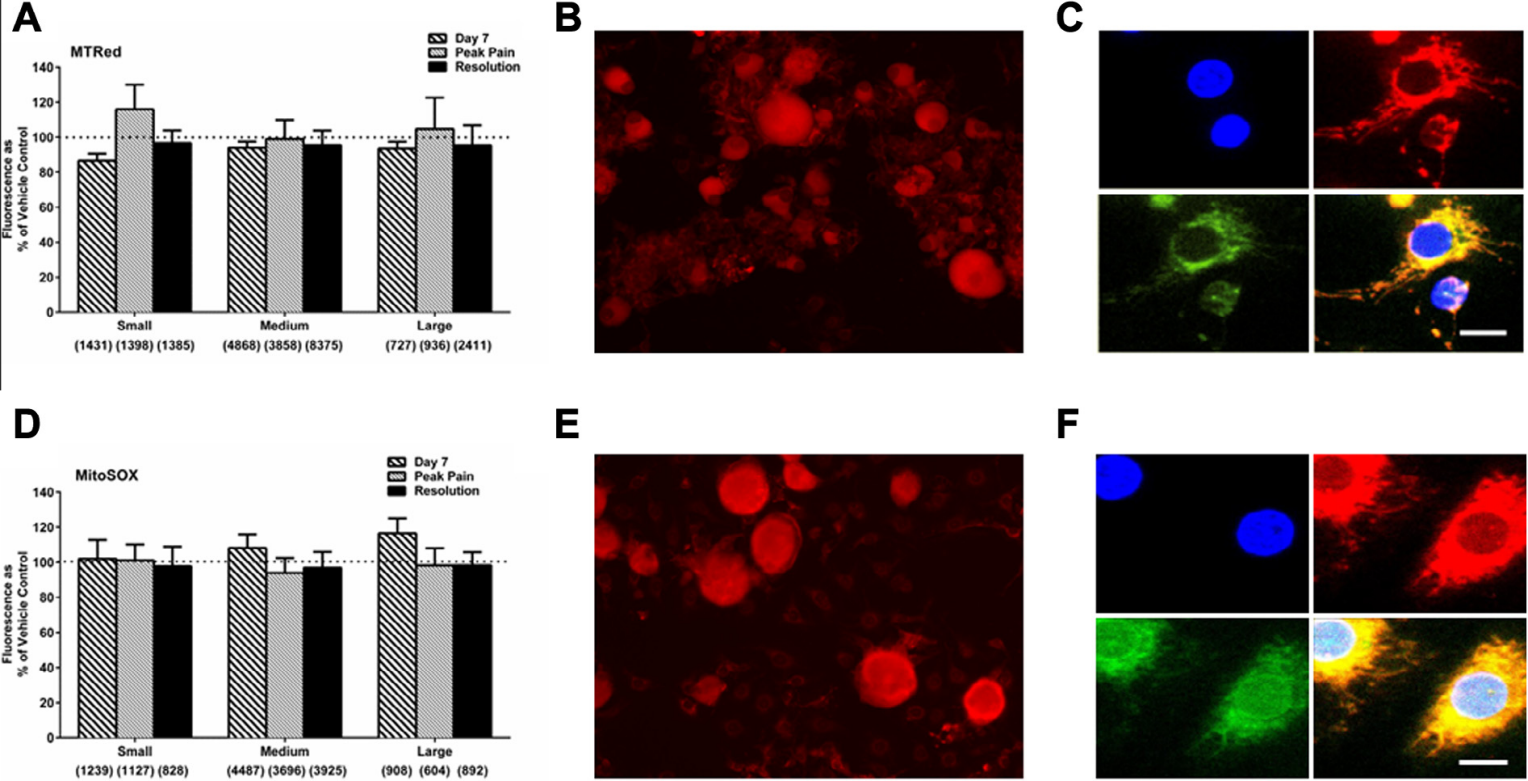
Effect of *in vivo* paclitaxel on total ROS and superoxide production in isolated DRG neurons. (A) The mean ± SEM of MTRed fluorescence intensity in small, medium and large isolated DRG neurons from paclitaxel-/vehicle-treated rats at day 7, peak pain (day 23–31) and pain resolution (day 173–194), *n* = 7–8 animals per treatment group. Neuronal categories were based on cell body size; small (10–25 μm), medium (25–50 μm) and large (>50 μm). Numbers in brackets indicate total number of neurons analysed in each category from paclitaxel- and vehicle-treated rats combined. Dotted line indicates the fluorescence of the vehicle-control group set as 100%. (B) Representative image of MTRed staining in an isolated DRG preparation. (C) MTRed colocalisation in non-neuronal DRG cells; DAPI stained nuclei are shown in blue, MitoTracker Red and MitoTracker Green are shown in red and green, respectively. Scale bar = 10 μm. (D) The mean ± SEM of MitoSOX fluorescence intensity of intensity in small, medium and large isolated DRG neurons from paclitaxel-/vehicle-treated rats at day 7, peak pain (day 24–31) and pain resolution (day 173–194), *n* = 7–8 per treatment group. Numbers in brackets indicate total number of neurons analysed in each category from paclitaxel- and vehicle-treated rats combined. Dotted line indicates the fluorescence of the vehicle-control group set as 100%. (E) Representative image of MitoSOX staining in an isolated DRG preparation. (F) MitoSOX Red colocalisation in non-neuronal DRG cells; DAPI stained nuclei are shown in blue, MitoSOX Red and MitoTracker Green are shown in red and green, respectively. Scale bar = 10 μm.

Atypical mitochondria were previously observed in both peripheral sensory nerves and DRG of rats prior to and during paclitaxel-induced pain (Flatters and Bennett, 2006, Jin et al., 2008, Barriere et al., 2012). As mitochondria are a major source of ROS, we examined ROS levels in isolated DRG neurons from paclitaxel- and vehicle-treated rats at day 7, peak pain and pain resolution, using MTRed and MitoSOX reduced probes. There was no significant change in the levels of total ROS (Fig.2A) or superoxide (Fig.2D) in small, medium or large DRG neurons from paclitaxel-treated rats compared to size-matched DRG neurons from vehicle-treated rats at any time-point. In separate experiments, *in vitro* paclitaxel exposure (250 ng/ml – previously shown to induce mitochondrial dysfunction (Melli et al., 2008)) did not significantly alter total ROS or superoxide levels in isolated naive DRG neurons compared to controls (data not shown).

As ROS levels were unaltered, we confirmed our experimental approach with additional experiments. Firstly, we confirmed MTRed and MitoSOX are entering mitochondria under our experimental conditions, as our primary DRG cultures co-labelled with either probe in combination with MitoTracker® Green, showed clear co-localisation of fluorescence (Fig.2C, F). Secondly, additional experiments using 500 nM MTRed also showed no significant difference in MTRed fluorescence of DRG neurons from paclitaxel-treated rats compared to those of vehicle-treated rats at day 7. The mean fluorescence of DRG neurons with 500 nM MTRed from paclitaxel-treated rats expressed as a percentage of mean fluorescence of DRG neurons from vehicle-treated rats was 103.9%, 106.7% and 93.6%, for small, medium and large neurons, respectively (*n* = 5 animals per group). This indicates that the lack of difference in DRG ROS levels between paclitaxel- and vehicle-treated rats is not due to saturation of the probe at 5 μM. These data suggested that the essential axotomy/enzymatic processing required to isolate DRG neurons, elevated ROS levels and thereby masked any alteration in ROS levels from a direct result of paclitaxel treatment *in vivo*.

**Fig. 3:**
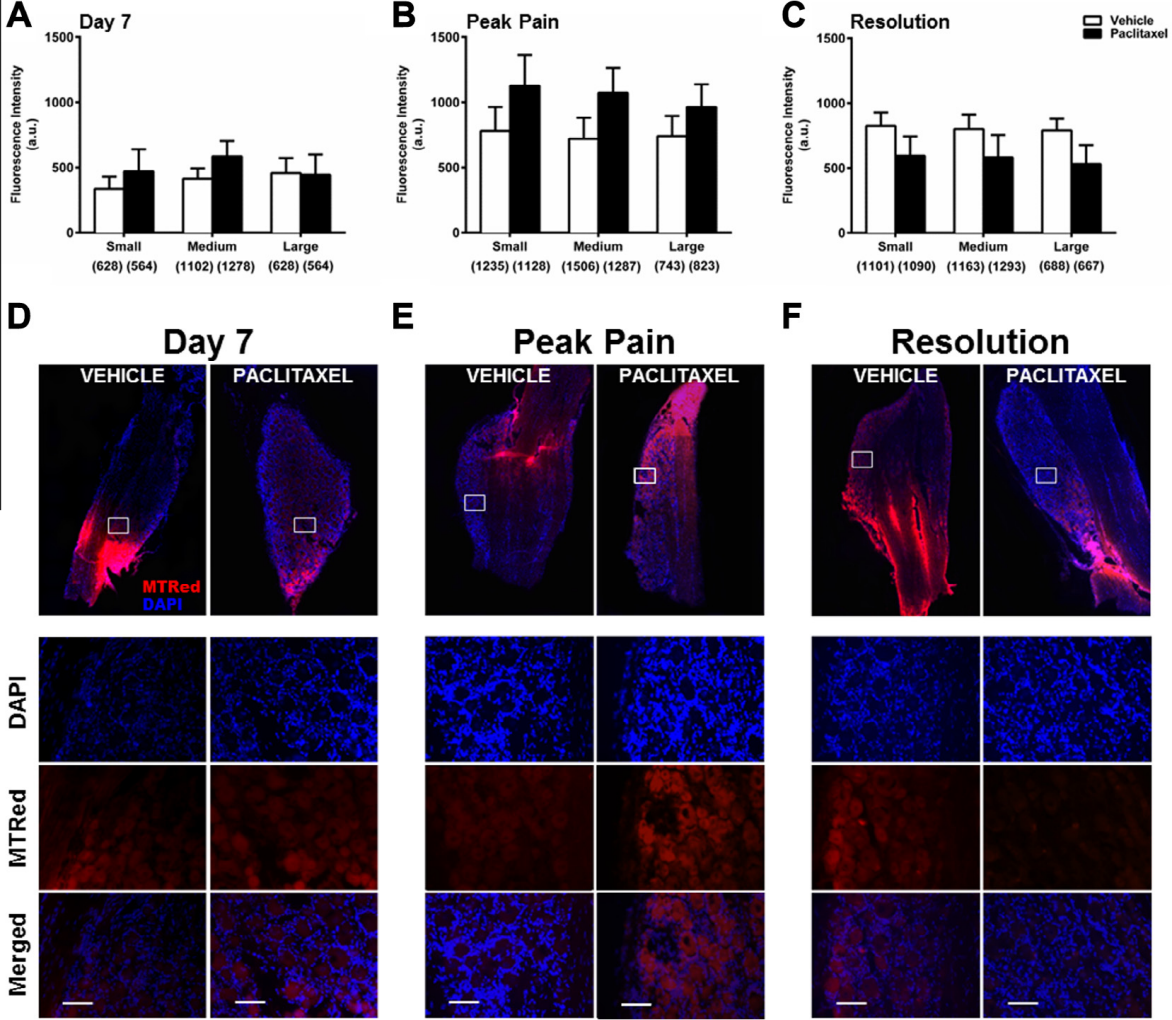
*In vivo* ROS levels in DRG neurons during the time-course of paclitaxel-induced painful neuropathy. (A–C) The mean ± SEM of MTRed fluorescence intensity of small, medium and large DRG neurons in paclitaxel- and vehicle- treated rats *in vivo* at (A) day 7, (B) peak pain – day 27–30 and (C) pain resolution – day 195–220. Neuronal categories were based on cell body size; small (10–23 μm), medium (23–32 μm) and large (>32 μm). Numbers in brackets indicate total number of neurons counted in each category, *n* = 6 animals per treatment group, per time point. (D–F) Example images of cut DRG from each treatment group; (D) day 7, (E) peak pain and (F) pain resolution. White boxed areas are highlighted to show DAPI, MTRed and merged image channels. Scale bar = 50 μm. Areas of intense MTRed staining are due to proximity of tissue to intrathecal injection site and were avoided during analysis.

**Fig. 4:**
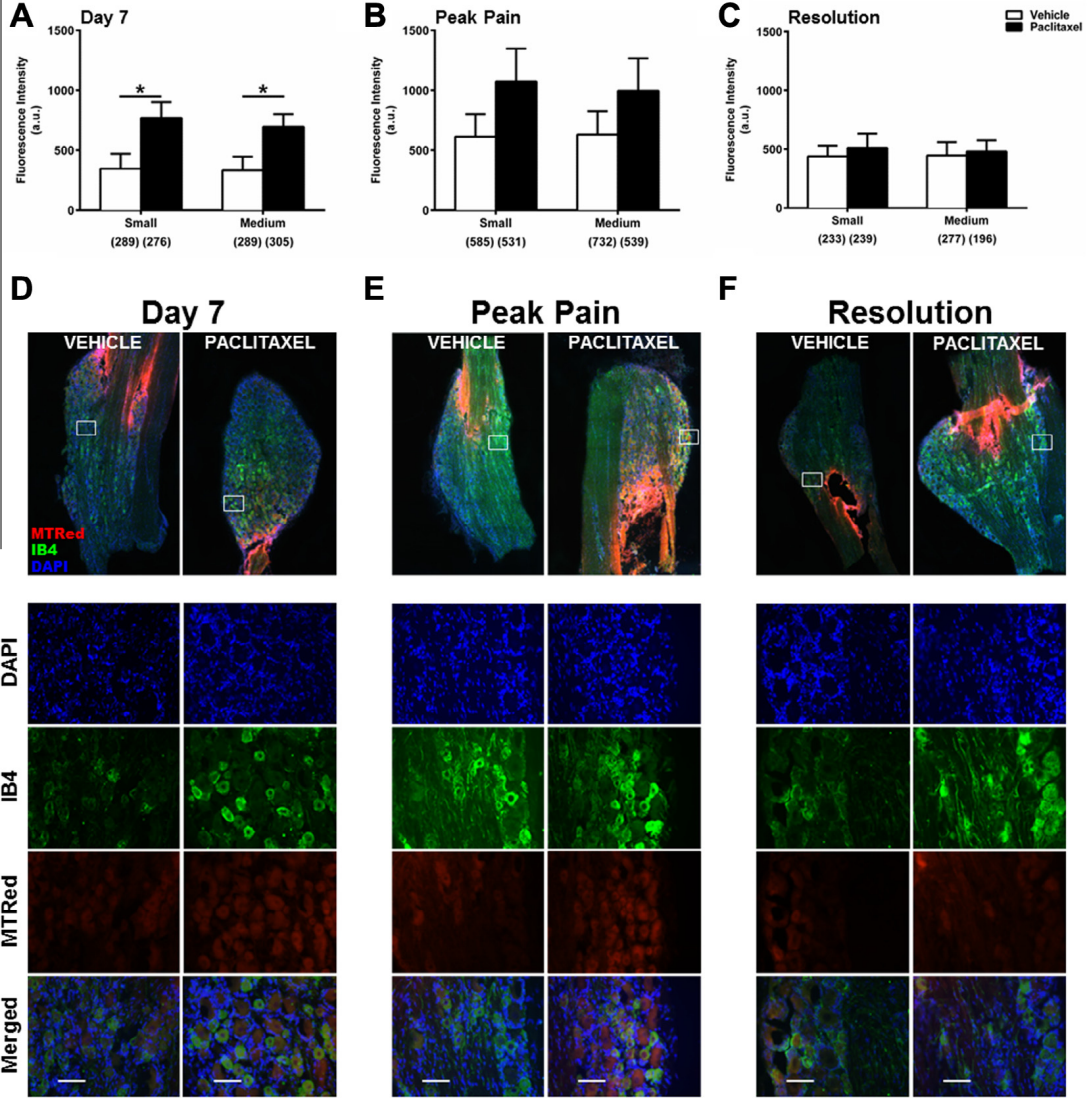
*In vivo* ROS levels in IB4+ DRG neurons during the time-course of paclitaxel-induced painful neuropathy. (A–C) The mean ± SEM of MTRed fluorescence intensity of small and medium IB4+ DRG neurons in paclitaxel- and vehicle-treated rats *in vivo* at (A) day 7, (B) peak pain – day 27–30 and (C) pain resolution – day 195–220. Neuronal categories were based on cell body size; small (10–23 μm) and medium (23–32 μm). Numbers in brackets indicate total number of neurons counted in each category, *n* = 5–6 animals per treatment group, per time point. ^*^p < 0.05 unpaired, one-tailed *t*-tests. (D–F) Example images of cut DRG from each treatment group; (D) day 7, (E) peak pain and (F) pain resolution. White boxed areas are highlighted to show DAPI, IB4+, MTRed and merged image channels. Scale bar = 50 μm. Areas of intense MTRed staining are due to proximity of tissue to intrathecal injection site and were avoided during analysis.

To examine ROS levels *in situ* during the time-course of paclitaxel-induced painful neuropathy, paclitaxel-/vehicle-treated rats received intrathecal injections of MTRed/vehicle *in vivo*. DRG and spinal cord were then analysed to determine ROS levels in neuronal/cellular populations. Small increases in ROS levels were seen in small and medium neurons at peak pain (Fig.3B), but were not statistically significant. Overall, there was no significant change in ROS levels of DRG neurons *in vivo* during the paclitaxel-induced pain time-course (Fig. 3). However, trends for altered levels of ROS production in DRG neurons led us to investigate whether specific subpopulations of neurons had altered ROS levels. There was no significant difference in ROS levels of peptidergic (CGRP+) neurons in paclitaxel-treated rats, compared to those in vehicle-treated rats, at day 7 and peak pain (data not shown). However, at pain resolution, ROS levels were significantly decreased in small peptidergic neurons of paclitaxel-treated rats compared to those in vehicle-treated rats (data not shown). Loss of peptidergic neurons is unlikely as the proportion of CGRP+ neurons is similar in paclitaxel- and vehicle-treated rats at all time-points (data not shown). In comparison, ROS levels were increased in IB4+ neurons in paclitaxel-treated rats at day 7 and peak pain compared to vehicle-treated. At day 7, significant increases in ROS levels in both small (122%) and medium-sized DRG neurons (110%) in paclitaxel-treated rats were observed (Fig. 4). At peak pain, ROS levels were increased by 74% in small neurons and 57% in medium neurons, but these increases were not statistically significant (Fig.4B). At pain resolution, ROS levels were unaltered in IB4+ neurons of paclitaxel-treated rats, compared to vehicle-treated rats (Fig.4C). Gain of non-peptidergic neurons is unlikely as the proportion of IB4+ neurons is similar in paclitaxel- and vehicle-treated rats at all time-points (data not shown).

**Fig. 5:**
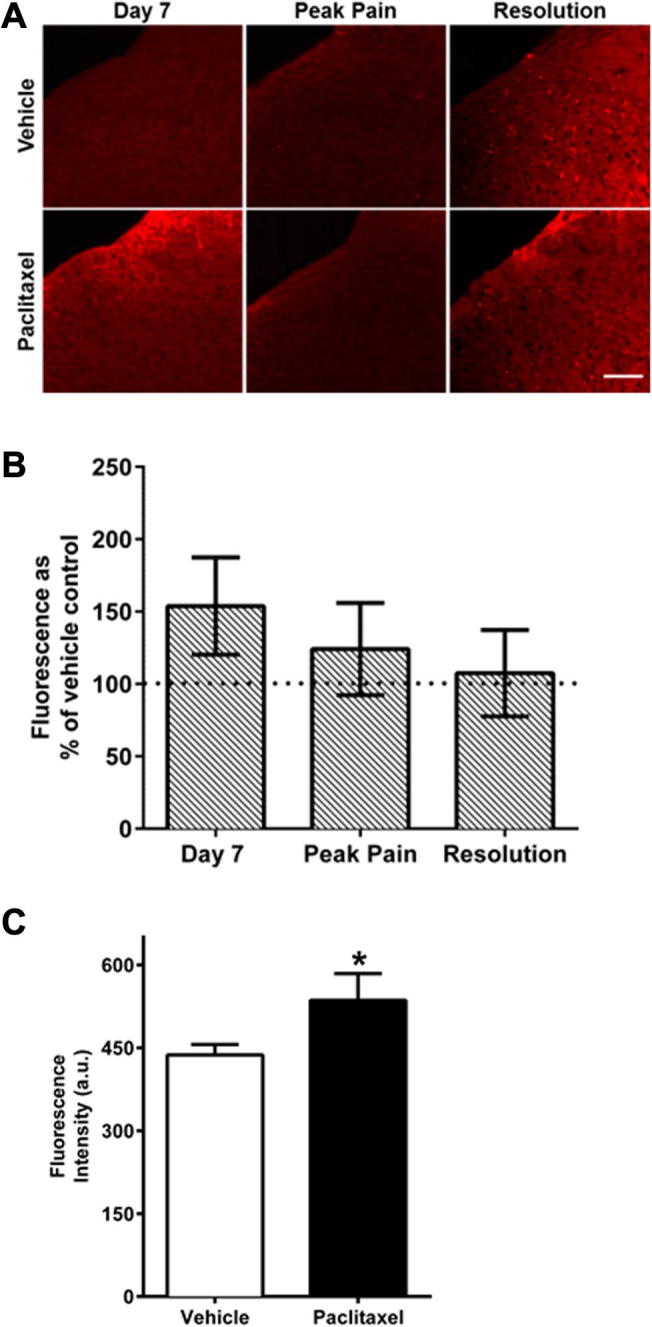
*In vivo* ROS levels in the superficial dorsal horn of the spinal cord during the time-course of paclitaxel-induced painful neuropathy. (A) Example images of superficial dorsal horn in sections of L4/5 spinal cord, following intrathecal injection of MTRed. Scale bar = 50 μm. (B) The mean ± SEM of MTRed fluorescence intensity in laminae I–III of L4/5 spinal cord in paclitaxel-treated animals, expressed as a percentage of vehicle-treated controls at day 7, peak pain (day 27–30) and pain resolution (day 195–220). *n* = 6–9 animals per treatment group, per time-point. (C) The mean ± SEM of MTRed fluorescence intensity of NeuN positive neurons in the dorsal horn of vehicle- and paclitaxel-treated rats at day 7. ^*^p < 0.05 unpaired, one-tailed *t*-test, *n* = 5–7 animals per group. No significant difference in ROS levels of spinal microglia or astrocytes in paclitaxel-treated rats compared to vehicle-treated rats was evident at day 7, *n* = 4 animals per group (data not shown).

Fig. 5 shows *in vivo* ROS levels in laminae I–III of the spinal cord during the time-course of paclitaxel-induced painful neuropathy, in paclitaxel-/vehicle-treated rats at day 7, peak pain and pain resolution time-points. Compared to vehicle-treated rats, spinal ROS levels were only increased at day 7, although this increase was not statistically significant (Fig. 5A, B). Further analysis examined if ROS levels at day 7 were increased in specific cellular subpopulations within laminae I–III in paclitaxel-treated rats. ROS levels within NeuN-positive cell bodies were significantly increased by 23% in paclitaxel-treated rats compared to vehicle-treated rats (Fig.5C). In contrast, there was no significant difference in ROS levels of spinal microglia and GFAP fluorescence had zero percent co-localisation with MTRed fluorescence in paclitaxel-/vehicle-treated rats at day 7 (data not shown).

**Fig. 6:**
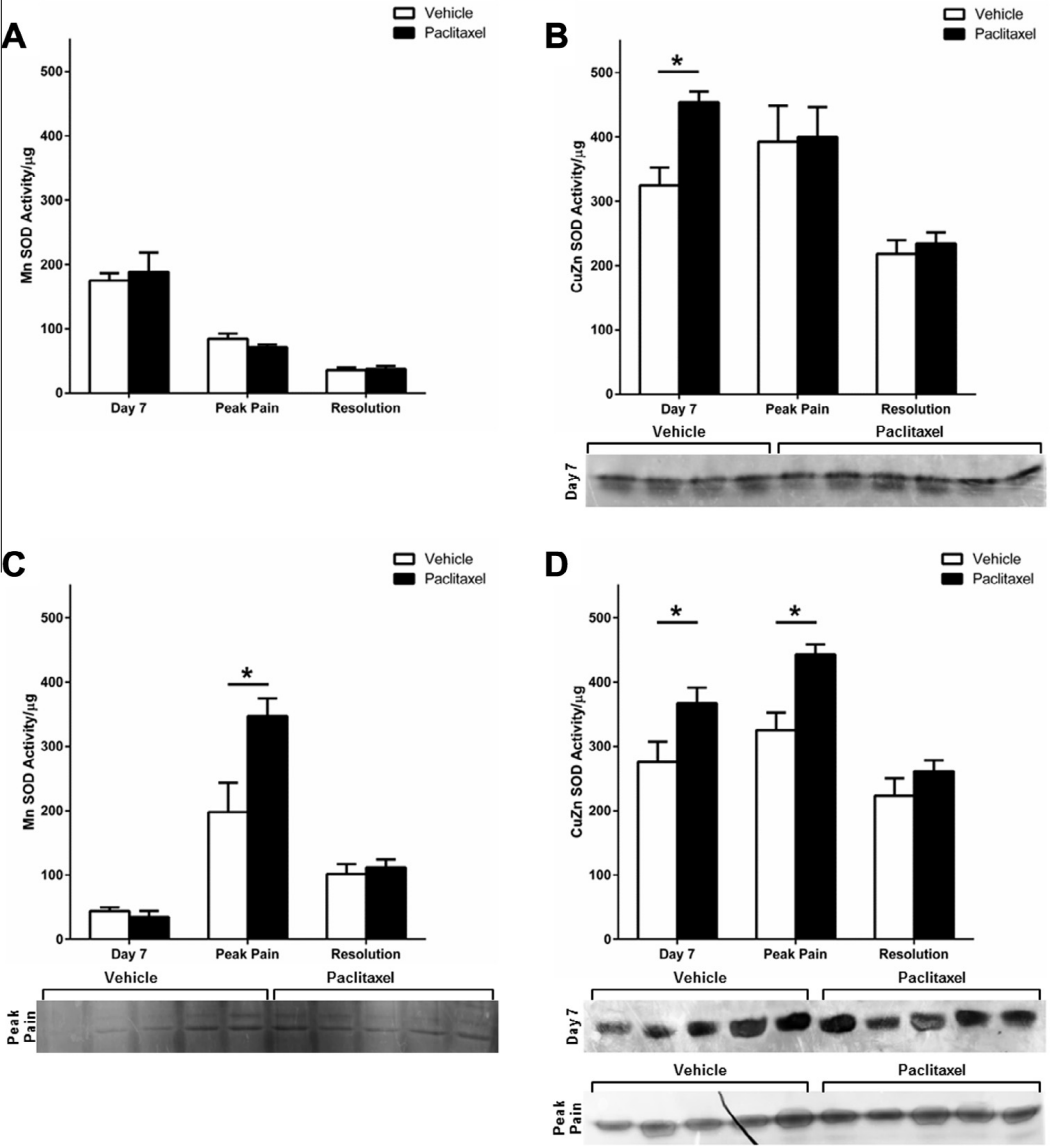
Superoxide dismutase activity in the DRG and saphenous nerve during the time-course of paclitaxel-induced painful neuropathy. MnSOD activity in (A) DRG and (C) saphenous nerves of paclitaxel- and vehicle-treated rats at day 7, peak pain (day 28) and pain resolution (day 182–218), *n* = 4–6 animals per group, ^*^p < 0.05 unpaired, two-tailed *t*-tests. CuZnSOD activity in (B) DRG and (D) saphenous nerves of paclitaxel- and vehicle-treated rats at day 7, peak pain (day 28) and pain resolution (day 182–218), *n* = 4–6 animals per group, ^*^p < 0.05 unpaired, two-tailed *t*-test. Adjacent panels display scans of activity gels used in analysis, which show significantly altered activity between groups. Black line on peak pain gel due to small tear.

After determining *in vivo* paclitaxel exposure increased ROS levels in DRG and spinal neurons; we investigated the activity of antioxidant enzyme activity during the time course of paclitaxel-induced pain in DRG and saphenous nerves. We measured activity of two subtypes of SOD: manganese superoxide dismutase (MnSOD), and copper zinc superoxide dismutase (CuZnSOD) and observed the expected approximate ratio of 3:1 (MnSOD:CuZnSOD) (Nedeva et al., 2004) in both DRG and saphenous nerves from paclitaxel-/vehicle-treated rats throughout the time-course. There was no significant difference in MnSOD activity between paclitaxel- and vehicle-treated rats in the DRG at any time-point (Fig.6A). In comparison, there was a significant 40% increase in CuZnSOD activity in DRG from paclitaxel-treated rats at day 7 compared to DRG from vehicle-treated rats (Fig.6B). CuZnSOD activity in the DRG was unaltered in paclitaxel-treated rats at peak pain and pain resolution time-points. In the saphenous nerve at day 7, there was no change in MnSOD activity (Fig.6C); however there was a significant 33% increase in CuZnSOD activity in paclitaxel-treated rats compared to vehicle-treated rats (Fig.6D). By peak pain, activities of MnSOD and CuZnSOD in the saphenous nerve were both significantly increased, 76% and 36% respectively, in paclitaxel-treated rats, compared to vehicle-treated rats (Fig.6C, D). At pain resolution, MnSOD and CuZnSOD activity were unaltered in saphenous nerves of paclitaxel-treated rats (Fig.6C, D).

**Fig. 7:**
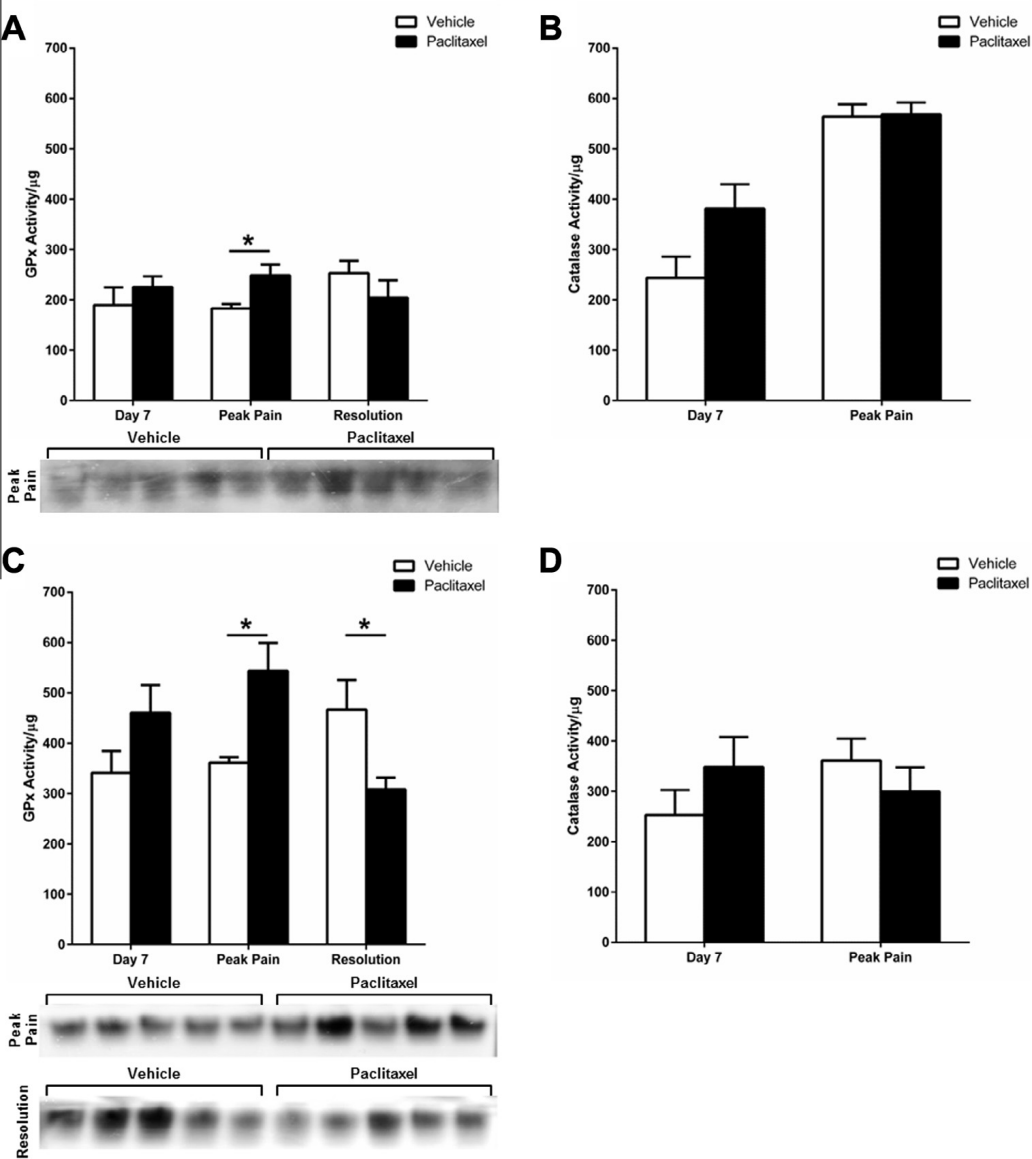
Glutathione peroxidase (GPx) and catalase activity in the DRG and saphenous nerve during the time-course of paclitaxel-induced painful neuropathy. GPx activity in (A) DRG and (C) saphenous nerves of paclitaxel- and vehicle-treated rats at day 7, peak pain (day 28) and pain resolution (day 182–218), *n* = 4–6 animals per group. (B) Catalase activity in the DRG of paclitaxel- and vehicle-treated rats at day 7 and peak pain (day 28), *n* = 3–5 animals per group. (D) Catalase activity in saphenous nerves of paclitaxel- and vehicle-treated rats at day 7 and peak pain (day 28), *n* = 5 animals per group. ^*^p < 0.05 unpaired, two-tailed *t*-tests. Adjacent panels display scans of activity gels used in analysis, which show significantly altered activity between groups.

Fig. 7 shows the activity of GPx and catalase in DRG and saphenous nerves during the time-course of paclitaxel-induced painful neuropathy. In the DRG, there was a significant 36% increase in GPx activity in paclitaxel-treated rats at the peak of paclitaxel-induced pain, but no change at day 7 and pain resolution, compared to concurrent vehicle-treated groups (Fig.7A). Similarly, in saphenous nerves, there was a significant 51% increase in GPx activity in paclitaxel-treated rats at the peak of paclitaxel-induced pain compared to the concurrent vehicle-treated group (Fig.7C). There was a 35% increase in GPx activity at day 7 in saphenous nerves from paclitaxel-treated rats, but this was not statistically significant. At pain resolution, there was a significant 34% decrease in GPx activity in saphenous nerves from paclitaxel-treated rats compared to vehicle-treated rats (Fig.7C). Catalase activity was not significantly altered in DRG or saphenous nerves from paclitaxel-treated rats at day 7 and peak pain time-points (Fig.7B, D), therefore was not assessed at pain resolution.

## Discussion

Our previous preclinical studies demonstrated that paclitaxel-induced painful neuropathy is associated with increased atypical mitochondria in sensory axons (Flatters and Bennett, 2006). Furthermore, pharmacological scavenging of ROS inhibited development and maintenance of the paclitaxel-induced pain syndrome *in vivo* (Fidanboylu et al., 2011). Here, for the first time, we have directly investigated the effect of paclitaxel on ROS levels in sensory neurons *in vitro* and *in vivo*. Moreover, these studies have assessed levels of ROS and antioxidant enzyme activity in the nociceptive system, prior to (day 7), during (peak pain), and at resolution of paclitaxel-induced painful neuropathy. *In vitro* exposure of naive DRG neurons to paclitaxel did not alter mitochondrial-derived ROS or mitochondrial superoxide levels. The concentration of paclitaxel (250 ng/ml) and exposure time (3 h) used, were the same as a previous study reporting mitochondrial dysfunction in isolated DRG neurons following *in vitro* paclitaxel exposure (Melli et al., 2008). DRG neurons isolated from paclitaxel-treated rats prior to, during and at resolution of paclitaxel-induced pain, did not show any change in mitochondrial ROS or superoxide levels compared to DRG neurons from vehicle-treated rats. This was unexpected, given previous evidence of mitochondrial dysfunction in DRG neurons following *in vivo* paclitaxel administration (Barriere et al., 2012). We suggest the reason for unaltered ROS/superoxide levels in isolated DRG neurons observed here, is due to the essential axotomy and enzymatic dissociation of DRG neurons, elevating ROS levels to such an extent that paclitaxel-induced changes in ROS are not detectable in this experimental setting.

Following intrathecal administration of reduced ROS probe MTRed, *in vivo* ROS levels both within DRG neurons and the spinal cord could be quantified *in situ*. In small and medium DRG neurons, increased ROS levels were indicated at day 7 and peak pain time-points, but these increases were not statistically significant when examining entire neuronal populations categorised by cell diameter only. However, when subpopulations of nociceptive neurons were identified immunohistochemically and analysed separately; clear differential effects were observed between paclitaxel-/vehicle-treated rats at the three time-points of interest. At day 7, there was more than a twofold increase in ROS levels in non-peptidergic IB4+ neurons from paclitaxel-treated rats whereas ROS remained unaltered in peptidergic CGRP+ neurons. At peak pain, ROS was increased in IB4+ neurons from paclitaxel-treated rats whereas ROS levels were unaltered in peptidergic CGRP+ neurons. At pain resolution, ROS levels in IB4+ neurons did not change, but were reduced in peptidergic CGRP+ neurons from paclitaxel-treated rats. Recently, evidence has shown paclitaxel increases MAPK levels differentially in CGRP+ neurons compared to IB4+ neurons during paclitaxel-induced pain (Li et al., 2015). Our data suggest that paclitaxel preferentially evokes ROS in non-peptidergic neurons prior to the pain syndrome, which persists in this neuronal subpopulation to peak pain severity, but resolves when the pain disappears. Thus, indicating ROS upregulation in IB4+ neurons specifically contributes to development of paclitaxel-induced pain and perhaps, to a lesser extent, the maintenance of the pain syndrome. Ablation of the central terminals of IB4+ primary afferents following intrathecal IB4-saporin markedly inhibited the development of oxaliplatin-induced pain (Joseph et al., 2008). In addition, both paclitaxel- and oxaliplatin-induced pain behaviours were inhibited by antioxidants (Flatters et al., 2006, Joseph et al., 2008, Fidanboylu et al., 2011). Collectively these data suggest that paclitaxel- and oxaliplatin-induced painful neuropathy may share a similar ROS driven, IB4+ neuron-dependent causal mechanism. In addition, paclitaxel-induced mechanical hypersensitivity was attenuated when TRPA1 channel activity was blocked through pharmacological antagonism (Chen et al., 2011) or genetic manipulation (Materazzi et al., 2012). TRPA1 channels are a known neuronal ROS sensor (Andersson et al., 2008) and are predominantly expressed on IB4+ DRG neurons (Barabas et al., 2012). Therefore, the preferential elevation of ROS in IB4+ neurons described here could suggest a direct mechanism of TRPA1 channel activity contributing to paclitaxel-induced pain. However, further experiments are required to directly assess this potential mechanism. In the superficial dorsal horn of the spinal cord, *in vivo* ROS levels were elevated at day 7 only, prior to the emergence of paclitaxel-induced pain. Further analysis on spinal cellular subpopulations at day 7 identified ROS was significantly increased in neurons, but unaffected in microglia and astrocytes, indicating that neuronal-derived ROS in the spinal cord contributes to the development of paclitaxel-induced painful neuropathy.

Previous studies examined distribution of paclitaxel following systemic administration (Lesser et al., 1995, Cavaletti et al., 2000, Xiao et al., 2011). Paclitaxel was present in liver, but not nervous tissues (spinal cord, DRG, sciatic nerve, brain), 2 h following a single administration of [^3^H]-paclitaxel (Lesser et al., 1995). In comparison, 24 h following repeated systemic administration (cumulative dose of 25 mg/kg paclitaxel), high levels were found in the DRG (Cavaletti et al., 2000) and smaller levels at other sites: sciatic nerve ⩾ liver > spinal cord > brain & plasma. At day 7, using the same dosing regimen as here (cumulative dose of 8 mg/kg paclitaxel), high levels were found in liver and DRG, with smaller levels at other sites: ventral root > dorsal root > sciatic nerve > spinal cord > brain & plasma (Xiao et al., 2011). Paclitaxel was only present in the DRG and liver at day 16, 10 days following the last 2 mg/kg dose of paclitaxel (Xiao et al., 2011). In relation to our data, these studies suggest that increased ROS levels observed at day 7 in DRG and spinal cord could be attributed to direct action of paclitaxel at these sites; perhaps via binding to β-tubulin associated with voltage-dependent anion channel (VDAC) subunits found on the mitochondrial membrane (Carre et al., 2002, Kidd et al., 2002). The persistence of paclitaxel in the DRG, albeit at much lower levels, may also be responsible for smaller elevation in ROS seen at the peak pain time point.

Collectively, distribution studies suggest that the majority of the neurotoxic damage caused by paclitaxel is directed at the DRG and peripheral sensory nerves. The net effect of paclitaxel-induced ROS, and its causality to pain, is likely to be somewhat reliant on how ROS is managed by endogenous antioxidant enzymes. In mammalian cells, mitochondria are not the only source of ROS; peroxisomes, endoplasmic reticulum, cytoplasm and the extracellular space are all sites at which ROS is produced (see (Brown and Borutaite, 2012)). Here, we analysed CuZnSOD/MnSOD and GPx/catalase activity, to appreciate the management of superoxide and hydrogen peroxide, respectively – prior to, during, and at resolution of paclitaxel-induced pain. The response of the antioxidant system during the time-course of paclitaxel-induced painful neuropathy differs between DRG and saphenous nerves (purely sensory nerves) when compared to similar tissues from vehicle-treated rats. In DRG, there was a significant increase in CuZnSOD and GPx activity, at day 7 and peak pain, respectively. MnSOD and catalase activity in the DRG were unaltered throughout the time-course of pain behaviour. In saphenous nerves from paclitaxel-treated rats at day 7; GPx activity is slightly increased; CuZnSOD activity significantly increased; and MnSOD/catalase activity unaffected. At peak pain, activity of all antioxidant enzymes was elevated in saphenous nerves, except catalase. At pain resolution, paclitaxel-evoked increases in antioxidant enzyme activity in saphenous nerves had also resolved. The increased MnSOD and CuZnSOD activity in saphenous nerves during paclitaxel-induced pain observed here, differ to a previous report describing decreased MnSOD and unchanged CuZnSOD activity at a similar time-point (Janes et al., 2013). The reason for this difference is unclear, perhaps due to differing techniques.

Electron transport chain activity results in elevation of superoxide in both the mitochondrial matrix and mitochondrial intermembrane space. We have found an upregulation in MnSOD and CuZnSOD activity in paclitaxel-treated rats. MnSOD is found exclusively in the mitochondrial matrix. Increased MnSOD activity was only evident in saphenous nerves at the peak pain time-point in this study. This indicates that the accumulation of paclitaxel immediately following injection (Xiao et al., 2011), is not sufficient to directly increase MnSOD activity *in vivo,* despite paclitaxel-exposure being shown to induce MnSOD mRNA expression *in vitro* (Das et al., 1998). In comparison, CuZnSOD is typically found in the cytosol. However, CuZnSOD has been shown to associate with the outer mitochondrial membrane (Nishiyama et al., 1996) and to localise to the inner mitochondrial membrane using metallochaperone CCS1 (copper chaperone for SOD) (Okado-Matsumoto and Fridovich, 2001, Sturtz et al., 2001, Kawamata and Manfredi, 2008). CCS1 acts at the inner membrane to metallate SOD, promoting CuZnSOD folding and retention in the intermembrane space (Sturtz et al., 2001, Bihlmaier et al., 2008, Kawamata and Manfredi, 2008). Therefore, it is possible that elevated CuZnSOD activity in paclitaxel-treated rats occurs in response to increased mitochondrially-located superoxide. Alternatively, elevated CuZnSOD activity could indicate increased cytosolic superoxide. Cytosolic superoxide following paclitaxel may be generated in mitochondria. As binding of paclitaxel to β-tubulin will result in pore opening in the mitochondrial membrane (Varbiro et al., 2001, Carre et al., 2002, Kidd et al., 2002), this provides a means for mitochondrial superoxide to leak from the mitochondrial matrix into the cytosol. In addition, paclitaxel has also been shown to prevent the closure of the mitochondrial permeability transition pore (PTP) via stabilising mitochondrial-microtubule interactions, thus potentially maintaining this superoxide leak (Evtodienko et al., 1996). Such events would ultimately cause swelling and vacuolisation of mitochondria, as previously observed in the saphenous nerves and DRG of paclitaxel-treated rats (Flatters and Bennett, 2006, Barriere et al., 2012). It was not possible to directly quantify ROS levels in saphenous nerves due to technical limitations. However, the increased activity of endogenous antioxidant enzymes in saphenous nerves at day 7 and peak pain time points indicate elevated ROS levels – a likely consequence of atypical mitochondria in saphenous nerves observed at these time points (Flatters and Bennett, 2006).

Although GPx activity increased at day 7 and peak pain, catalase activity remained unaltered in DRG and saphenous nerves. Unaltered catalase activity indicates a moderate level of hydrogen peroxide manageable via GPx’s high affinity for the substrate; catalase is known to function only at high concentrations due to low hydrogen peroxide affinity, and a decrease in catalase activity would indicate levels of hydrogen peroxide great enough to deactivate the catalytic moiety (Altomare et al., 1974, Halliwell and Gutteridge, 2015). This functional information, in conjunction with GPx’s ability to locate close to the site of hydrogen peroxide production; both mitochondrially and cytosolically (Halliwell and Gutteridge, 2015), demonstrate the important labile nature of GPx in managing hydrogen peroxide. GPx can prevent activation of peroxisome-bound catalase, supporting the concept of mitochondrially-derived ROS in paclitaxel-induced painful neuropathy, with GPx preventing hydrogen peroxide-induced cell death. Lack of degeneration and cell death in the DRG in this low-dose model has been previously established (Flatters and Bennett, 2006).

A number of changes in antioxidant activity are concurrent between DRG and saphenous nerves i.e. CuZnSOD increase at day 7 and GPx increase at peak pain. Other changes – increases in MnSOD/CuZnSOD activity at peak pain – are only observed in saphenous nerves. This implies that ROS elevation in saphenous nerves is greater and/or prolonged compared to DRG at peak pain, or that the existing antioxidant activity capacity in DRG is greater than in saphenous nerves. Increased SOD activity at day 7 in DRG and saphenous nerves indicates a subsequent need to manage elevated hydrogen peroxide; which drives the upregulation of GPx activity we observe at peak pain time-point. We suggest there is an inadequate antioxidant response at day 7, resulting in elevated ROS prior to pain onset. This causes further mitochondrial dysfunction (indicated by continued elevated SOD at peak pain) and/or initiation of signalling cascades that underlie the coasting phenomenon to peak pain severity at day 28. Indeed, our recent work has highlighted the importance of early events in the genesis of this pain state; as antimycin A (complex III inhibitor) inhibited development of paclitaxel-induced pain only when administered before & during paclitaxel administration (Griffiths and Flatters, 2015).

## Conclusions

This is the first study to identify *in vivo* ROS within sensory neurons in conjunction with the development and maintenance of paclitaxel-induced painful neuropathy. Thus linking ROS in peripheral and central axons to the propagation of paclitaxel-induced pain. The response of the endogenous antioxidant system appears to be inadequate and delayed in its onset. We suggest that this causes excessive ROS, which enhances pain signalling, leading to the development of persistent pain.
